# Usefulness of Pelvic Radiographs in the Initial Trauma Evaluation with Concurrent CT: Is Additional Radiation Exposure Necessary?

**DOI:** 10.1155/2018/6260954

**Published:** 2018-10-02

**Authors:** Anne K. Misiura, Autumn D. Nanassy, Jacqueline Urbine

**Affiliations:** ^1^Hahnemann University Hospital, 230 North Broad Street, Philadelphia, PA 19102, USA; ^2^St. Christopher's Hospital for Children, 160 East Erie Avenue, Philadelphia, PA 19134, USA

## Abstract

Trauma patients in a Level I Pediatric Trauma Center may undergo CT of the abdomen and pelvis with concurrent radiograph during initial evaluation in an attempt to diagnose injury. To determine if plain digital radiograph of the pelvis adds additional information in the initial trauma evaluation when CT of the abdomen and pelvis is also performed, trauma patients who presented to an urban Level I Pediatric Trauma Center between 1 January 2010 and 7 February 2017 in whom pelvic radiograph and CT of the abdomen and pelvis were performed within 24 hours of each other were analyzed. A total of 172 trauma patients had pelvic radiograph and CT exams performed within 24 hours of each other. There were 12 cases in which the radiograph missed pelvic fractures seen on CT and 2 cases in which the radiograph suspected a fracture that was not present on subsequent CT. Furthermore, fractures in the pelvis were missed on pelvic radiographs in 12 of 35 cases identified on CT. Sensitivity of pelvic radiograph in detecting fractures seen on CT was 65.7% with a 95% confidence interval of 47.79-80.87%. Results suggest that there is no added diagnostic information gained from a pelvic radiograph when concurrent CT is also obtained, a practice which exposes the pediatric trauma patient to unnecessary radiation.

## 1. Introduction

Pelvic radiographs are utilized to identify fractures and dislocations which, with the exception of small avulsion fractures, are uncommon in the pediatric population. Pelvic fractures in children, other than avulsion fractures, result from a high energy mechanism of injury such as being struck by a motor vehicle. When more than minor injury is suspected in these instances, computed tomography (CT) of the abdomen and pelvis is the most helpful for diagnosis of bony traumatic findings [1–3]. Currently, pelvic radiograph is recommended routinely for pediatric patients with altered level of consciousness or other distracting injuries which may result in lower reliability of the physical examination of the pelvis [4]. When significant injury to the pelvis is suspected, CT will often be performed due to additional suspected injury in the abdomen. But in cases when CT is not planned, the current pediatric emergency medicine textbooks recommend a single anterior-posterior radiograph of the pelvis if indicated by physical examination [5, 6]. A review by Guillamondegui et al. of 130 pediatric pelvic fractures found that only 54% of fractures were identified by pelvic radiographs alone, which calls into question the utility of the pelvic radiograph [7].

Additionally, CT of the abdomen and pelvis provides much more information about soft tissue injury that is lacking on radiographs. Given the mechanisms of injury in pediatric pelvic fractures, soft tissue injury should be suspected. Bond et al. reviewed 2,248 pediatric patients with blunt trauma, of which 54 had bony injury to the pelvis [8]. Results from this study, along with a larger review of 16,630 pediatric and adult trauma registry patients by Demetriades et al., suggest that the location and severity of the fracture are strongly associated with the probability of abdominal injury [9]. While 6% of isolated pubic fractures had concomitant soft tissue injury, soft tissue injury was seen in 33% of ileal or pelvic rim fractures and in 80% of cases with multiple pelvic fractures [8].

Observationally in our radiology department at an urban Level I Pediatric Trauma Center in the northeastern United States, a significant number of pediatric trauma patients who underwent CT of the abdomen and pelvis in the initial trauma evaluation period also had pelvic radiographs performed. Given the risk of soft tissue damage in mechanisms of injury in pediatric trauma patients, CT tests are necessary and frequently ordered. The purpose of our study is to determine if a plain digital radiograph of the pelvis adds further information when CT of the abdomen and pelvis is also performed in the pediatric trauma evaluation and to calculate the sensitivity of identifying fractures subsequently diagnosed by CT.

## 2. Materials and Methods

Institutional Review Board approval was obtained for this retrospective data analysis prior to the commencement of the study. Retrospective reviews of imaging reports were performed on all trauma patients in the emergency department at an urban Level I Pediatric Trauma Center in the northeastern region of the United States, from 1 January 2010, when the Picture Archiving and Communication System (PACS) system was first utilized at our institution, to 17 February 2017. Patients who had both a pelvic radiograph and CT of the abdomen and pelvis performed during the initial evaluation or within 24 hours of each other were identified and included in analyses. Patients with CT of the abdomen and pelvis who only underwent radiographs following open reduction and internal fixation (ORIF) were not included, as these were postoperative evaluations.

Reports of the studies read by board certified pediatric radiologists were reviewed to identify findings related to the pelvis. The pelvic findings of each patient on CT and radiograph were compared to identify discrepancies. The sensitivity and specificity of pelvic plain digital radiograph in identifying fractures were determined using CT as the referenced standard. Clinical information collected from the patients included age, sex, reported mechanism of injury, and timing of ordering and performing imaging studies when applicable.

## 3. Results

A total of 172 patients met inclusion criteria in the specified time period. Of the 172 patients, 71 were females and 101 were males. Patient age ranged from 1 month to 19 years, with a mean age of 9.84 years and standard deviation of 4.89 years. Mechanisms of injury are outlined in Table 1. The three most common mechanisms of injury were automobile versus pedestrian collisions, falls, and motor vehicle crashes.

There were no cases in which the radiograph interpretation provided additional information not initially provided on the CT interpretation. Bony traumatic findings were present in 20% of patients (*n* = 35). There was no discrepancy between CT and radiograph interpretation related to these bony pelvic findings in 23 of these 35 patients. However, in the remaining 12 cases, some or all of the bony pelvis findings were not included on the radiograph interpretation when compared to the CT interpretation. There were two patients in which the radiograph interpretation was positive (concern for subluxed sacroiliac joint and sacroiliac joint fracture), but a CT obtained later was negative, indicating false positive radiographs. As shown in Table 2, sensitivity of radiographs in identifying fractures was 65.7% with a 95% confidence interval of 47.79-80.87%, with specificity, positive predictive value, and negative predictive value of 98.5%, 92.0%, and 91.8%, respectively.

The average time between CT and radiograph acquisition was 123 minutes but ranged from 5 minutes to 16 hours. In approximately 65% of cases, the studies were performed within 60 minutes of each other and in 42% of cases within 30 minutes of each other.

## 4. Discussion

**Figure 1:**
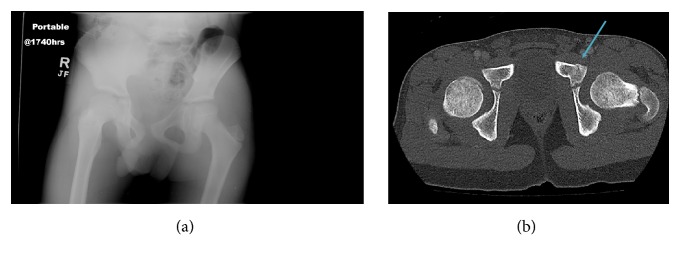
A portable radiograph of the pelvis (a) was performed on a 7-year-old male who was the victim of an automobile versus pedestrian trauma. No fracture or dislocation is seen on the radiograph. Twenty-nine minutes later, a contrast-enhanced CT of the abdomen and pelvis (b) was performed on the same patient demonstrating a nondisplaced left superior pubic ramus fracture (arrow).

Retrospective analysis of trauma patients at an urban Level I Pediatric Trauma Center revealed that there was no information gained with the additional diagnostic test of pelvic radiograph against CT interpretation, and plain digital pelvic radiographs had less than desirable sensitivity in picking up fractures/subluxations (see Figure 1).

**Figure 2:**
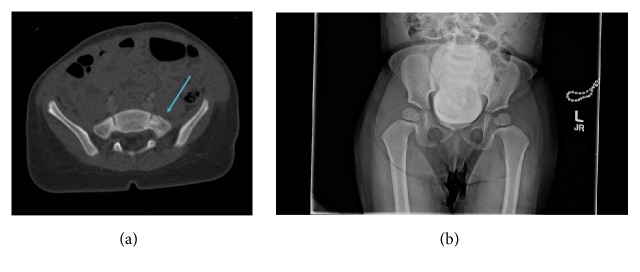
A 2-year-old female presented as a Level 1 Trauma; a contrast-enhanced CT of the abdomen and pelvis (a) was performed, revealing a left sacral fracture (arrow). Subsequently, a radiograph of the pelvis (b) did not demonstrate the same finding, secondary to marked bladder distention following contrast administration for the CT.

Our results also demonstrate that fractures are missed almost half the time on pelvic radiograph, which is complementary to Guillamondegui et al.'s aforementioned study in 2003, despite advances in imaging over the last decade. The quality and techniques of our study are more consistent than the cohort in Guillamondegui's as all of our studies were performed at a single institution, and our conclusions are more general to all pelvic bony trauma as we included all pelvic bony trauma rather than focusing on certain regions. Further, in addition to information collected in Guillamondegui et al.'s study, our data provides added context related to mechanisms of injury surrounding presentation. Results suggest that, in cases of clinically suspected pelvic fractures, radiographs should not be performed during the initial trauma evaluation, nor after a CT has already been performed (see Figure 2). Although plain digital pelvic radiographs have high specificity, they are lacking in sensitivity that CTs can provide.

In the case of one patient included in the analysis, a pelvic radiograph was obtained during active cardiopulmonary resuscitation as part of group of portable radiographs which would guide immediate clinical decision-making. When the patient was stabilized, he then underwent CT of the head, neck, chest, abdomen, and pelvis. In cases like this, it is necessary to obtain radiographs first to gain helpful information during resuscitation, because CT is not feasible.

Radiographs are not benign tests; pediatric radiation doses are difficult to generalize due to the wide range of patient size from neonate to teenager. The average adult dose of pelvic radiographs is 0.7 mSv. This is equivalent to roughly 1/13 of the radiation of a CT of the abdomen and pelvis, which in an adult is about 10 mSv [10]. In one study by Miglioretti et al., data on CTs in the pediatric population across six integrated healthcare systems were analyzed. The study calculated a mean radiation dose for CT abdomen and pelvis of 10.6 mSv in children below 5 years of age to 14.8 mSv among children ranging from 10 to 14 years [11]. Radiation exposure is well known to increase the risk of malignancy and possibly other noncancer diseases, and pediatric population is more susceptible to these risks than adults [12, 13]. Miglioretti et al. projected 1 radiation-induced solid cancer to result from 300-390 abdomen and pelvis CTs in girls and from 670-760 CTs in boys [11]. It is important to keep risks associated with radiation in mind when imaging children.

The retrospective data collected from a seven-year period of time at a single urban Level I Pediatric Trauma Center provide a wide variability in patient age and mechanism of injury, which increases the generalizability of results to other pediatric populations. However, pelvic fractures are not frequently seen in the pediatric population, and the sample size of positive findings is small. The order of the study acquisition may skew the data, though this is most likely in cases when the pelvic radiograph was obtained after the CT interpretation was available, which would result in higher sensitivity of detecting fractures given the availability of the CT report at the time of radiographic interpretation. The majority of cases of CT and radiograph obtained within one hour of each other were actually ordered by the clinician before either one was performed. In some of these cases, it is possible that the duplicate diagnostic imaging was not ordered intentionally. For example, the triage personnel may have initially ordered a plain digital pelvic radiograph; however after trauma physician evaluation, a CT of the abdomen and pelvis may have been determined to be necessary, but the initial pelvic radiograph order was never cancelled.

It is important to note that while we discuss the role of imaging in the urgent traumatic patient, less critically ill patients with lower suspicion for pelvic injury may benefit from nonemergent MRI evaluation on a case-by-case basis. This imaging workup may also be more appropriate for subacute trauma patients who present to the emergency department days after their injury. One limitation, however, is that MRI is not always available 24/7 and may result in unnecessary delays in diagnosis due to prolonged wait times. Appropriate triage is crucial.

Our results are novel and generalizable to other pediatric trauma centers. Providers should strive to not duplicate or order unnecessary tests whenever possible because these practices can result in undue radiation exposure and wasted resources. When placing a new order, providers should be diligent in reviewing outstanding orders and results already returned to avoid any unnecessary radiation exposure. In the future, studying the impact of electronic order entry on superfluous medical imaging in the Level I Trauma Center setting may elucidate other tests that result in unnecessary radiation to the pediatric patient.

## 5. Conclusion

Radiographs are redundant to the pediatric trauma patient who undergoes CT and may result in unnecessary radiation exposure. Care should be taken not to duplicate studies during trauma evaluation in the pediatric patient.

## Figures and Tables

**Table 1:** Mechanism of injury in pediatric trauma patients.

Mechanism	*n*
AVP	84
Fall	28
MVC	27
Hit by falling object	3
NAT	2
ATV accident	2
Bicycle accident	2
BAT	2
MBC	2
Found down	1
Football injury	1
Assault	1
Gunshot	1
Not specified	16
Total (*N*)	172

AVP: Automobile vs. pedestrian, MVC: Motor-vehicle collision, NAT: Non-accidental trauma, ATV: All-terrain vehicle, BAT: Blunt abdominal trauma, MBC: Motor-bike collision.

**Table 2:** Results of plain digital pelvic radiographs in pediatric trauma patients.

Parameter	Bony traumatic findings (*n*)
FN	12
FP	2
TN	135
TP	23
*N*	172
Sensitivity	65.71% (95% CI: 47.79-80.87%)
Specificity	98.54% (95% CI: 94.83-99.82%)
Accuracy	91.86%
NPV	91.84% (95% CI: 87.67-94.68%)
PPV	92.00% (95% CI: 74.00-97.89%)

FN: False negative, FP: False positive, TN: True negative, TP: True positive, N: total number of radiographs, NPV: Negative predictive value, PPV: Positive predictive value. Overall sensitivity and specificity were 65.71% and 98.54%, respectively, with overall accuracy of 91.86%. The positive predictive value was 92.00% and the negative predictive value was 91.84%.

## Data Availability

The medical record data used to support the findings of this study are restricted by the Drexel University Human Research Protection Program in order to protect patient privacy.
